# *Hemolivia* species infecting Central American wood turtles (*Rhinoclemmys pulcherrima manni*) and problems with differential diagnosis within the genus *Hemolivia*[Fn FN1]

**DOI:** 10.1051/parasite/2023067

**Published:** 2024-02-14

**Authors:** Žaneta Živčicová, Jana Kvičerová, Pavel Široký

**Affiliations:** 1 Department of Biology and Wildlife Diseases, Faculty of Veterinary Hygiene and Ecology, University of Veterinary Sciences Brno Palackého 1946/1 61242 Brno Czech Republic; 2 Department of Parasitology, Faculty of Science, University of South Bohemia Branišovská 1760 37005 České Budějovice Czech Republic; 3 Department of Zoology, Faculty of Science, Charles University, Biocev Průmyslová 595 252 50 Vestec Czech Republic; 4 CEITEC – Central European Institute of Technology, University of Veterinary Sciences Brno Palackého 1946/1 612 42 Brno Czech Republic

**Keywords:** Hemogregarine, *Hemolivia*, Nicaragua, Differential diagnosis, Morphology

## Abstract

Blood parasites of the genus *Hemolivia* Petit, Landau, Baccam and Lainson, 1990 (Adeleorina: Karyolysidae) are hemogregarines of ectothermic vertebrates, such as lizards, chelonians, and toads. Only five species of *Hemolivia* from vertebrate hosts and one from their tick vector have been described so far. In the present study, Central American wood turtles (*Rhinoclemmys pulcherrima manni*) originating from Southern Nicaragua were screened for the presence of hemogregarines. Ten out of 30 specimens (33.3%) were positive for *Hemolivia* using both approaches – microscopy and PCR-based analyses. Phylogenetic analyses based on the 18S rRNA gene revealed the presence of two haplotypes, both placed as sister taxa in the *Hemolivia* clade. Their phylogenetic position was supported by high bootstrap values and high posterior probabilities, suggesting that there are at least two new distinct haplotypes corresponding to two distinct species. However, the specimens of each haplotype were microscopically indistinguishable from each other based on the gamont morphology, therefore, only a single species could be described and named, as *Hemolivia pulcherrima* n. sp. We consider that the uniform morphology of the most common blood stages of species of the genus *Hemolivia* complicates their differential diagnosis. Sequence divergence and different host spectra, therefore, remain the only differentiating tools.

## Abstract (fr)

Les parasites sanguins du genre *Hemolivia* Petit, Landau, Baccam et Lainson, 1990 (Adeleorina : Karyolysidae) sont des hémogrégarines de vertébrés ectothermes, tels que les lézards, les tortues et les crapauds. Seules cinq espèces d’*Hemolivia* provenant d’hôtes vertébrés et une de leur tique vectrice ont été décrites jusqu’à présent. Dans cette étude, des tortues peintes d’Amérique centrale (*Rhinoclemmys pulcherrima manni*) originaires du sud du Nicaragua ont été examinées pour détecter la présence d’hémogrégarines. Dix tortues sur 30 (33,3 %) étaient positives pour *Hemolivia* en utilisant les deux approches de microscopie et d’analyse de PCR. Les analyses phylogénétiques basées sur le gène de l’ARNr 18S ont révélé la présence de deux haplotypes, tous deux placés comme taxons frères dans le clade *Hemolivia*. Leur position phylogénétique était étayée par des valeurs de bootstrap et des probabilités postérieures élevées, suggérant qu’il existe au moins deux nouveaux haplotypes distincts correspondant à deux espèces distinctes. Cependant, les spécimens de chaque haplotype étaient impossibles à distinguer les uns des autres au microscope sur la base de la morphologie des gamontes. Par conséquent, une seule espèce a pu être décrite et nommée, comme *Hemolivia pulcherrima* n. sp. Nous considérons que l’uniformité de la morphologie des stades sanguins les plus courants des espèces du genre *Hemolivia* complique leur diagnostic différentiel. Les divergences de séquences et les différents spectres d’hôtes restent donc les seuls outils de différenciation.

## Introduction

The genus *Hemolivia* Petit, Landau, Baccam and Lainson, 1990 (Coccidia: Adeleorina: Karyolysidae) contains tick-transmitted hemogregarines parasitizing ectothermic vertebrates. The biology of these parasites differs from other closely related genera of hemogregarines. Sporogony is divided into two phases; the first phase comprises formation of oocyst with sporokinetes, and in the second phase sporocyst and sporozoites are formed. Merogony is intraerythrocytic. Gamonts of *Hemolivia* occur in peripheral blood of vertebrates inside a stain-resistant parasitophorous vacuole. At this stage, they are easily morphologically recognizable from other hemogregarine genera by their elliptical bar shape with a straight long axis and a blue-stained nucleus located in the polar position [11, 15, 28].

Six species of *Hemolivia* have been described so far. The type species, *Hemolivia stellata* Petit, Landau, Baccam et Lainson, 1990 was first described infecting Cane toad (*Rhinella marina*) and teiid lizard *Ameiva ameiva* in the Neotropical region [12, 19]. *Hemolivia mariae* Smallridge et Paperna, 1997 is a species infecting Australian skinks *Tiliqua rugosa* and *Egernia stokesii* [26, 27]. Three species of *Hemolivia* have been described from chelonian hosts: *Hemolivia mauritanica* (Sergent et Sergent, 1904) infecting Palearctic tortoises *Testudo graeca* and *T. marginata* [1, 13, 17, 20–22], *Hemolivia parvula* (Dias, 1953) from the African tortoises *Kinixys zombensis* and *Stigmochelys pardalis* [3], and *Hemolivia cruciata* Zechmeisterová et Široký, 2022 infecting Vietnamese box turtle *Cuora galbinifrons* [31]. *Hemolivia argantis* (Garnham, 1954) originating from Egypt was first described as *Hepatozoon argantis* from the soft tick *Argas brumpti*. Based on morphology of its developmental stages in the definitive host it was reassigned by Karadjian *et al*. [9] to the genus *Hemolivia*. Nevertheless, the vertebrate host, DNA sequence as well as morphology of blood stages of *H. argantis* are unknown, which makes this species hardly comparable to other *Hemolivia* species [9].

This study aimed to investigate the samples from Central American wood turtles *Rhinoclemmys pulcherrima manni* from Southern Nicaragua containing yet undescribed *Hemolivia* parasites, and to compare them with other *Hemolivia*. We used a combination of morphological characteristics and 18S rRNA gene sequences to infer its evolutionary relationships and phylogeny.

## Material and methods

### Ethics

The turtles were not part of any experimental work. The blood sampling was carried out in accordance with the valid Czech legislation, *i.e*., Act on Veterinary Care and Amendment of Certain Related Acts (Veterinary Act) no. 166/1999 Sb.

### Sampling

Thirty pet-traded Central American wood turtles (*R. pulcherrima manni*) originating from Southern Nicaragua were sampled during veterinary screening in the Czech Republic in March 2013. Blood samples were collected by puncture of the dorsal caudal vein using insulin syringes equipped with fine needles. A drop of each blood sample was used to prepare a blood smear for microscopic analysis, and the remaining blood was fixed in 96% ethanol and stored in sterile microtubes at −20 °C for later molecular analyses.

### Microscopy

Blood smears were air-dried, fixed in absolute methanol for 10 min, stained with Giemsa as described in Široký *et al*. [23], and then microscopically examined with an Olympus BX53 microscope using the 100× magnification lens equipped with immersion oil. Photomicrographs were captured using an Olympus DP 73 digital camera. Morphometrical parameters were measured using the QuickPhoto Micro software 3.2 (Promicra, s.r.o., Prague, Czech Republic). Maximum length and width were recorded for the parasites, their nuclei, infected and uninfected erythrocytes, and their nuclei. All measurements are given in micrometers (μm) as the mean followed by standard deviation and range (in parenthesis). Furthermore, parameters LW (length × width) and L/W (length/width ratio) were calculated. The intensity of parasitemia was estimated for each infected turtle as the percentage of infected red blood cells found in approximately 10^4^ cells [23].

### DNA isolation, amplification, and sequencing

Collected blood samples were centrifuged, superfluous ethanol was removed, and remaining ethanol was evaporated. Genomic DNA was extracted from blood using the NucleoSpin Tissue kit (Macherey-Nagel, Duren, Germany), according to manufacturer’s protocol. Concentration of isolated DNA was measured on the Qubit 4 Fluorometer (ThermoFisher Scientific, Waltham, MA, USA). Three primer pairs targeting the 18S rRNA gene were used to detect *Hemolivia* parasites (Table 1). Since the commonly used HepF300/HepR900 primer pair covers only a short part of the 18S rRNA gene, we also applied the EF/ER and Hemo1/Hemo2 primer pairs to cover almost the whole gene length [31]. All PCR reactions were performed in 25 μL volume, including 1 μL of each primer, 12.5 μL of Combi PPP Master Mix (Top-Bio s.r.o., Prague, Czech Republic), 9.5 μL of PCR water (Top-Bio s.r.o.), and 1 μL of extracted DNA. PCR products were visualized on 1.5% agarose gel using the Midori Green (Elisabeth Pharmacon, Brno, Czech Republic) under UV light. All PCR products of expected sizes were purified using a Gel/PCR DNA Fragments Extraction Kit (Geneaid Biotech Ltd., New Taipei City, Taiwan). The purified PCR products were Sanger sequenced in both directions in a service laboratory (Macrogen Inc., Amsterdam, the Netherlands).

**Table 1:** Primers used in this study.

Primer name	Primer sequence 5′ → 3′	PCR conditions °C/s			No. of cycles	Length of PCR product [bp]
		Denaturation	Annealing	Extension		
EF	GAAACTGCGAATGGCTCATT	92/45	55/45	72/90	35	1500
ER	CTTGCGCCTACTAGGCATTC					
HepF300	GTTTCTGACCTATCAGCTTTCGACG	94/45	61/45	72/60		581
HepR900	CAAATCTAAGAATTTCACCTCTGAC					
Hemo1	TATTGGTTTTAAGAACTAATTTTATGATTG	94/45	51/45	72/90		900
Hemo2	CTTCTCCTTCCTTTAAGTGATAAGGTTCAC					

### Phylogenetic analyses

The taxonomic origin of the obtained sequences was verified by BLAST (https://blast.ncbi.nlm.nih.gov/Blast.cgi), and the sequences were assembled using the Sequence Scanner v1.0 (Applied Biosystems, Waltham, MA, USA) and DNASTAR v5.05 package (DNASTAR Inc., Madison, WI, USA). The obtained sequences were aligned together with additional sequences downloaded from the GenBank database (Supplementary Table S1); the alignment was created in BioEdit v7.0.5.3 [5], and manually trimmed to a uniform length. The final alignment was 1,525 bp long and contained 73 sequences. Phylogenetic reconstructions were performed using the Maximum Likelihood (ML) and Bayesian inference (BI) approaches, computed in PhyML v2.4.3 [4] and MrBayes v3.2.2 [8] programs, respectively. The best fitting model of evolution (GTR + Г + I) was calculated in SMS: Smart Model Selection (http://www.atgc-montpellier.fr/phyml-sms/). The ML was carried out with a non-parametric bootstrap analysis of 1,000 replicates. The BI was calculated with the Markov Chain Monte Carlo (MCMC) run for 10 million generations, and with tree sampling every 100 generations; the trees were summarized after removing 25% burn-in. The final phylogenetic trees were rooted and visualized in TreeView v1.6.6 [16], and graphically edited in Adobe Illustrator 2020 (Adobe Systems, Inc.).

## Results

### Microscopy

Hemogregarines of the *Hemolivia* morphology were detected in 10 out of 30 blood smears of examined Central American wood turtles (prevalence 33.3%). Mature gamonts predominated in eight blood smears, whereas a few earlier developmental stages – trophozoite, young gamonts, and stages thought to be merozoites or merozoites in transition to early merogony – were detected on the remaining two smears. The observed stages of parasites occurred only inside the erythrocytes. All gamonts were morphologically uniform with minor differences.

### Molecular analyses

All 30 samples were analyzed molecularly; 10 of them (33.3%) provided positive results. Sequences of a good quality were obtained from all PCR-positive blood samples. BLAST analysis of obtained sequences confirmed their identity with the genus *Hemolivia*. The sequences were identical with sequences of *Hemolivia* sp. (accession numbers KF992713 and KF992714) from *Rhinoclemmys pulcherrima* already published by Kvičerová *et al*. [11]. However, this *Hemolivia* sp. has not been named and described so far, supporting our assumption that the studied isolates represent a new species, not described so far.

**Figure 1:**
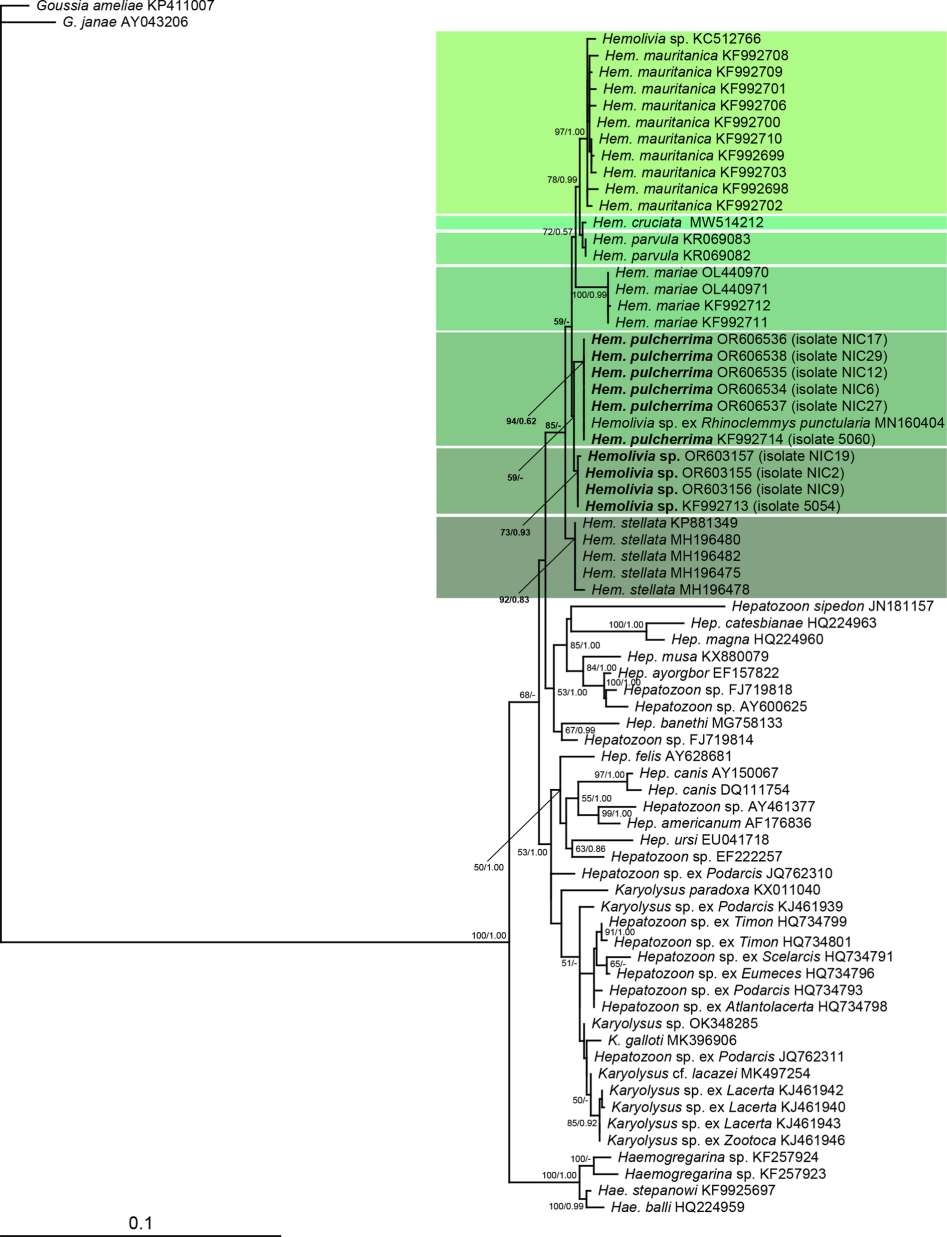
Phylogenetic relationships of *Hemolivia* species including *H. pulcherrima* n. sp. and *Hemolivia* sp. inferred by the ML analysis of 1,525 bp long part of the 18S rDNA sequences. Numbers at the nodes show bootstrap values derived from ML analysis/posterior probabilities under the BI analysis. Only bootstrap supports and posterior probabilities higher than 50% or 0.50, respectively, are displayed. *Goussia* was used as an outgroup. Taxa for which new sequences were obtained in this study are printed in bold.

Two distinct haplotypes of *Hemolivia* were revealed, clustering as sister taxa within the *Hemolivia* clade (Fig. 1). The first haplotype was represented by six isolates, whereas the second haplotype was represented by the remaining four isolates. Despite repeated sequencing, none of the obtained sequences indicated the pattern of co-infection (double peaks). Both haplotypes differed considerably (at seven nucleotide positions), while the observed difference in some cases delimited separate species (Supplementary Table S2). Nevertheless, the gamonts of both haplotypes were morphologically indistinguishable. Therefore, we used representatives of the more abundant haplotype for the description of a new species. Representatives of the second, less abundant haplotype, were analyzed in the same way, but separately.

Description of *Hemolivia* found in this study.

Apicomplexa Levine, 1980

Conoidasida Levine, 1988

Coccidia Leuckart, 1879

Adeleorina Léger, 1911

Family: Karyolysidae Wenyon, 1926

Genus: *Hemolivia* Petit, Landau, Baccam and Lainson, 1990.

### *Hemolivia pulcherrima* n. sp. (Fig. 2)

urn:lsid:zoobank.org:act:423D5BA3-6302-4B15-8CCE-8938EA0286E4

**Figure 2:**
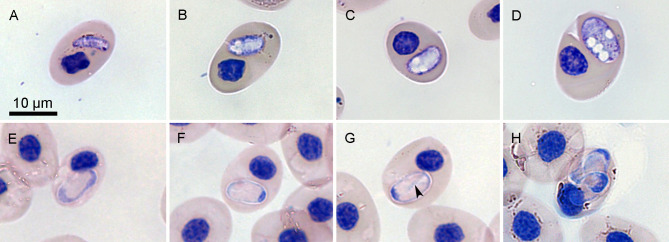
*Hemolivia pulcherrima* n. sp. in erythrocytes of the freshwater turtle *Rhinoclemmys pulcherrima manni*; all figures are in the same scale. Slim elongated stage resembling trophozoite (A). Merozoites in transition developing to meronts can be vacuolated (B, C). A single stage determined as possible meront contained large vacuoles, but no visible nuclei (D). Developing gamont in pre-final stage has a hardly visible or invisible nucleus (E). Mature gamont can have parallel arms in its parasitophorous vacuole (F), or crossed, visible as thin blue lines forming “X structure” (black arrowhead, G). Multiple infections of a single erythrocyte can be seen (H).

Type host: *Rhinoclemmys pulcherrima manni* (Dunn, 1930) (Testudines: Geoemydidae)

Other hosts: *Hemolivia* with identical 18S rDNA sequence (582 bp; GenBank MN160404) was detected by Nordmeyer *et al*. [14] from spot-legged turtle *Rhinoclemmys punctularia* (Daudin, 1801); therefore, we consider this turtle species to be another host of *Hemolivia pulcherrima* n. sp.

Vector: unknown

Type locality: Southern Nicaragua, exact locality unknown

Other localities: unknown

Prevalence and parasitemia: 6 out of 30 (20%) specimens of *R. pulcherrima manni* were positive. Parasitemia was 0.36 ± 0.29 (0.1–0.74).

Site of infection: Blood samples collected from live turtles; parasites detected in peripheral blood.

Type material: Blood smear and blood sample were deposited in collection of the Institute of Parasitology, Biology Centre of the Academy of Sciences of the Czech Republic, České Budějovice, Czech Republic, under the collection number IPCAS Pro 78.

Representative DNA sequence: DNA sample no. 5060 (1,421 bp long) deposited under accession number KF992714 is the representative of the type material.

Etymology: The specific epithet “*pulcherrima*” means “the most beautiful”. The authors are aware that the described species is not particularly beautiful, but to facilitate the work of future authors, we used the same euphonic specific epithet as the type host species.

Morphological description:

A single detected (tropho)zoite measured 8 × 3 μm, was slim, vacuolated, cucumber-shaped with one pointed end, its nucleus was not discernible (Fig. 2A). Merozoites were elongated, widely oval, measuring 8 × 4 μm (*n* = 3). Their cytoplasm contained vacuoles; nuclei were not detectable (Figs. 2B, 2C). A single early merogonic stage measured 10 × 6 μm. It had oval shape and possessed a vacuolated and heterogeneous cytoplasm (Fig. 2D). Gamonts of *Hemolivia pulcherrima* n. sp. were oval, slightly rounded and elongated, and were localized in a stain-resistant parasitophorous vacuole. Premature gamonts did not have distinct nuclei (Fig. 2E). Mature gamonts (Figs. 2F, 2G, 2H) resembled empty bars with oval or irregular shaped, dark purple-stained nucleus, located at the polar position, which is typical for the genus *Hemolivia*. The thin blue line along the long axis of the parasite indicates the curved position of the gamont inside the vacuole. These thin blue lines may be crossed in an “X” shape (Fig. 2G). The capsule measured 9.0 ± 0.7 (7–11) × 5.5 ± 0.7 (4–7) μm (*n* = 59), with LW 50.6 ± 9.2 (32–77) μm^2^ and L/W ratio 1.7 ± 0.2 (1.3–1.7). Nucleus of gamont measured 1.6 ± 0.5 (1–3) × 3.1 ± 0.5 (2–4) μm, with LW 4.8 ± 1.8 (2–9) μm^2^ and L/W ratio 0.5 ± 0.2 (0.25–1).

Effect on host cell: Infected blood cells were not affected by the presence of parasites, except for erythrocyte nuclei, which were pushed to the periphery in some cases. *Hemolivia* did not affect the staining of the host cell.

Remarks: Similar as other *Hemolivia* species, *Hemolivia pulcherrima* n. sp. had oval-shaped, straight gamonts, which belonged to shorter and more rounded *Hemolivia* species with smaller nucleus. Nevertheless, morphology and size of so far described parasites of this genus were quite uniform. That is why differential diagnosis of *Hemolivia* cannot be based solely on gamont morphology. However, some morphological differences have been described in *H. cruciata*, which can have noticeable folding lines, forming an “X” structure [31]. Nevertheless, our recent findings of this “X” in *H. pulcherrima* question the validity of this character. More prominent differences may be observed in geographical distribution and in molecular features of *Hemolivia* species. Parasites from chelonian hosts were recorded in Vietnam (*H. cruciata*), Mozambique (*H. parvula*), and in the Mediterranean and Middle East (*H. mauritanica*) [3, 6, 22, 24, 31]. There is one more report of *Hemolivia* in *Rhinoclemmys* turtle: Nordmeyer *et al*. [14] detected *Hemolivia* in a captive, but wild-caught *Rhinoclemmys punctularia* having an identical sequence with our *Hemolivia pulcherrima* n. sp. A list of all currently known species of *Hemolivia* together with their gamont sizes is reviewed in Table 2.

**Table 2:** List of *Hemolivia* species described so far.

Species name	Locality	Gamont size [μm]	Nucleus size [μm]	Type host	Other hosts	Vector	References
*Hemolivia argantis* (Garnham, 1954)	Egypt	–	–	Not known	Not known	*Argas brumpti*	[9]
*Hemolivia cruciata* Zechmeisterová et Široký 2022	Vietnam	11.9 ± 0.6 (11–13) × 5.9 ± 0.6 (5–7)	4.5 ± 0.9 (3–6) × 3.2 ± 0.7 (2–5)	*Cuora galbinifrons*	Not known	Not known	[31]
*Hemolivia mariae* Smallridge et Paperna, 1997	Australia	18 × 5	–	*Tiliqua rugosa*	*Egernia stokesii, Mabuya vittata, Agama stellio*	*Amblyomma limbatum*	[26–28]
*Hemolivia mauritanica* (Sergent et Sergent, 1904)	North Africa, Bulgaria, Greece, Turkey	12.5 × 5 (10–14 × 4–7)	–	*Testudo graeca*	*Testudo marginata*	*Hyalomma aegyptium*	[1, 13, 20, 21, 23]
*Hemolivia parvula* (Dias, 1953)	Mozambique	11–12.1 × 5.4–7.2; 12.1 ± 0.7 (10.3–3.2) × 5.6 ± 0.3 (4.7–6.1)	3.3 ± 0.5 (2.4–4.3) × 3.8 ± 0.4 (3.1–4.4)	*Kinixys zombensis*	*Stigmochelys pardalis*	Not known	[2, 3]
*Hemolivia pulcherrima* n. sp.	Southern Nicaragua	8.2 ± 0.8 × 4.9 ± 0.5; 9.0 ± 0.7 (7.0–11.0) × 5.5 ± 0.7 (4.0–7.0)	3.1 ± 0.6 × 1.6 ± 0.6; 1.5 ± 0.5 (1.0–2.0) × 3.1 ± 0.5 (2.0–4.0)	*Rhinoclemmys pulcherrima manni*	*Rhinoclemmys punctularia*	Not known	[11, 14]; this study
*Hemolivia stellata* Petit, Landau, Baccam et Lainson, 1990	Brazil	9 × 5.1	–	*Rhinella marina*	*Ameiva ameiva*	*Amblyomma rotundatum*	[9, 12, 19]

### *Hemolivia* sp. (Fig. 3)

Host species: *Rhinoclemmys pulcherrima manni* (Dunn, 1930) (Testudines: Geoemydidae)

**Figure 3:**
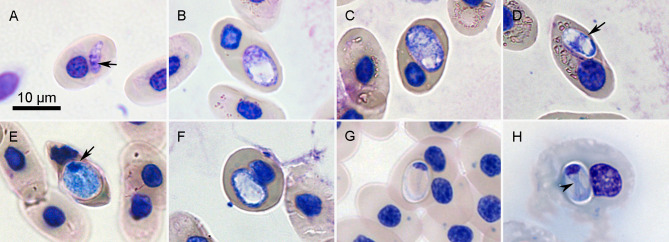
*Hemolivia* sp. in erythrocytes of the freshwater turtle *Rhinoclemmys pulcherrima manni*; all figures are in the same scale*.* Trophozoite is slim, cucumber-shaped, pointed towards one pole, and its cytoplasm contains small vacuoles. Nucleus (marked by black arrow) formed by granulated chromatin is placed sub-centrally (A). Developing meronts contain blue granulated or foamy cytoplasm without distinct nuclei (B, C). Young gamonts have nuclei in the centre (black arrow, D), their size grows, nucleus (marked by black arrow) heads towards one pole of parasitophorous vacuole (E), but sometimes is not visible (F). Mature gamonts have typical stain-resistant vacuole, nucleus in polar position (G, H), and their arms can be crossed forming a visible thin blue “X” (black arrowhead, H).

Vector: unknown

Locality: Southern Nicaragua, exact locality unknown

Prevalence and parasitemia: 4 out of 30 (13.3%) specimens of *R. pulcherrima manni* were positive. Parasitemia was 0.19 ± 0.7 (0.12–0.25).

Site of infection: Blood samples collected from live turtles; parasites detected in peripheral blood.

Material deposited: Blood smear and blood sample were deposited in collection of the Department of Biology and Wildlife Diseases, University of Veterinary Sciences Brno, Czech Republic, under the collection number NIC-4-13.

Representative DNA sequence: DNA sample no. 5054 (1,419 bp long) deposited in the GenBank database under accession number KF992714.

Morphological traits were the same as for *Hemolivia pulcherrima* n. sp. A single detected trophozoite measured 7 × 3 μm, was slim, vacuolated, cucumber-shaped with one pointed end, and possessed fragmented chromatin placed sub-centrally (Fig. 3A). Detected merogonic stages had vacuolated cytoplasm; nuclei were not detectable (Figs. 3B, 3C). Stages considered as young gamonts had foamy cytoplasm and nucleus either fragmented and placed at the centre of the developing gamont (Fig. 3D), concentrating towards one pole (Fig. 3E), or the nuclei were not visible (Fig. 3F). These young gamonts measured 9.4 ± 1.1 (8–11) × 4.9 ± 1.0 (4–7) μm (*n* = 8). The capsule of mature gamonts of the second haplotype representatives measured 8.6 ± 0.7 (7–10) × 5.4 ± 0.8 (3–7) μm (*n* = 29), with LW 46.8 ± 9.2 (24–63) μm^2^ and L/W ratio 1.6 ± 0.3 (1.3–2.7) (Figs. 3G, 3H). Nuclei of these gamonts measured 1.9 ± 0.5 (1–3) × 3.1 ± 0.6 (2–5) μm, with LW 6.1 ± 1.9 (3–10) μm^2^ and L/W ratio 0.7 ± 0.2 (0.3–1). Thin blue lines of curved mature gamonts were oriented along long axis of capsule (Fig. 3G), sometimes crossed in an “X” shape (Fig. 3H).

Effect on host cell was identical as described for *Hemolivia pulcherrima* n. sp.

Remarks: The only remarkable difference was detected by sequence analysis. Mature gamonts were identical as for *H. pulcherrima* n. sp. Young stages were generally more variable due to ongoing development in one of the blood smears. However, they were also not different from the usual *Hemolivia* development. Development in the invertebrate definitive host is unknown. At the moment, therefore, we can neither describe this isolate as a new species, nor synonymize it with other, already known species of *Hemolivia*.

## Discussion

*Hemolivia pulcherrima* n. sp. represents the sixth member of the genus *Hemolivia* with known stages from the vertebrate host, and with an available DNA sequence. These data allow its comparison and differential diagnosis with all other *Hemolivia* species except for *H. argantis,* so far described only by its morphology from the soft tick *Argas brumpti*. Nevertheless, this tick species has a geographically distant range from *H. pulcherrima*, being distributed in Eastern to Southern Africa, and also possessing a different host spectrum [7]. Therefore, we can exclude conspecificity of *H. pulcherrima* n. sp. with *H. argantis*. Currently, we have no reason to distrust the work of Karadjian *et al*. [9] and their classification of *H. argantis* in the genus *Hemolivia* and thus, we include it in list of valid *Hemolivia* species (Table 2). Rediscovery of *H. argantis* by new sampling and its molecular-genetic characterization would be useful to avoid uncertainties regarding differential diagnoses in future *Hemolivia* studies.

**Figure 4:**
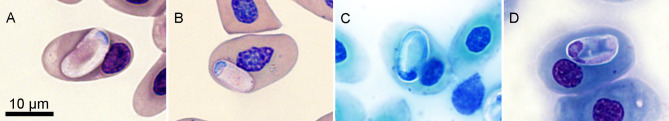
Morphological uniformity of *Hemolivia* gamonts; all figures are in the same scale. (A) *Hemolivia mauritanica* from *Testudo graeca*; (B) *H. mariae* from *Egernia stokesii*; (C) *H. parvula* from *Kinixys zombensis*; (D) *H. cruciata* from *Cuora galbinifrons*.

*Hemolivia* species so far described from vertebrate hosts are unique by the morphology of gamonts (Fig. 4), which is easily distinguishable from all other hemogregarine genera. The gamonts are not curved, but possess a straight, long axis; the parasitophorous vacuole is stain-resistant, making the gamont unstained, pale or whitish, with the nucleus being the only stained visible structure of the gamont, always placed in the polar position (Figs. 2F, 2G, 2H; Figs. 3G, 3H; Fig. 4). This feature and its uniformity easily distinguish *Hemolivia* from other genera of hemogregarines and thus may be beneficial for their easy generic classification. However, the mentioned morphological uniformity within the genus may become a problem preventing species differential diagnosis. Generally, the blood stages of most hemogregarines offer few reliable morphological traits useful for their morphological differentiation. Therefore, morphology became only a supplementary method in species diagnosis, as it is insufficient by itself.

Description of the life cycle of any hemogregarine is hardly possible only on the basis of one-time collected blood smears – a situation that is usual for most field collected wildlife. A blood smear captures the situation of a single moment, so it is usually dominated by mature gamonts. Obtaining information on the sequence of individual developmental stages in real time would require either experimental infection under laboratory conditions (*e.g*., for *Hemolivia*: [12, 18, 19, 23, 25]), or very frequent, repeated sampling of the same individuals in the field (*e.g*., using radiotelemetry). We named the detected blood stages by approximation with these *Hemolivia* species that have described life cycles.

Thus, description of new *Hemolivia* species can be quite difficult. Exceptions from the usual morphology are few [11, 31]; variability may be found in sizes of the parasites or their nuclei, or some variations of internal positions of gamonts like crossed lines in gamonts of *H. cruciata*, which create an “X” structure [31]. Nevertheless, the last-mentioned trait could be an artefact caused by pre-analytical sample treatment during a single study dealing with *H. cruciata*. Only future sampling of the same species can confirm its diagnostic reliability and stability, because the similar “X” structures have also been found in the present study on other *Hemolivia* species.

Since slight morphometric differences in a range of a single or few micrometers represent natural intraspecific variability or can be caused by dissimilar diagnostic approaches by various authors, this plays no role in differential diagnosis. To avoid creating synonymy with new described species, comparison of sequence data has become an inevitable part of alpha-taxonomy of hemogregarines.

Our study was based on 30 samples from Central American wood turtles (*R. pulcherrima manni*). In these turtles, we detected parasites that were morphologically identical with the genus *Hemolivia*. The morphology of blood stages and molecular features, supported by phylogeny and geography of turtle hosts, proved the record of a new species of this genus.

Molecular analyses were based on the 18S rRNA gene, which is commonly used to detect hemogregarines. The HepF300/HepR900 primer pair amplifies only its short fragment; therefore, we also used other complementary primer pairs. The primers EF/ER originally designed for *Eimeria* spp. can also be used for detection of other genera of hemogregarines and were proven to be useful in the genus *Hemolivia* [10, 11, 29, 31]. The primer pair Hemo1/Hemo2 is commonly used for detection of *Hepatozoon* spp. [2, 30]. Nevertheless, contrary to claims by Zechmeisterová *et al*. [31], amplification of the *Hemolivia* sequence using Hemo1/Hemo2 primer set was successful, which allowed us to obtain a longer sequence fragment from our isolates.

Phylogenetic analyses revealed two closely related haplotypes of the genus *Hemolivia*. The genetic distance of these haplotypes suggests two different conclusions: 1) significant 18S rDNA sequence variability within the species; 2) haplotypes of two closely related species. Because of the indistinguishable morphology of the parasites and the fact that our samples originate from the same host species and from the same area, we avoid description of the second isolate as a new, distinct species of *Hemolivia*. On the other side, sequences of both isolates differ to the scale usually justifying existence of distinct species (compare *e.g*., *H. cruciata* × *H. parvula*; Supplementary Table S2), and thus, we cannot consider both isolates, without any doubt, to be conspecific. Earlier analyses proposed that *H. pulcherrima* n. sp. might be a synonym of *H. stellata* since they can occur in the same region, and sequence data for *H. stellata* were unknown at that time [11]. However, our recent analyses showed that our isolates from *R. pulcherrima manni* represent clearly distinct species.

## Supplementary material

The supplementary material for this article can be found at https://www.parasite-journal.org/10.1051/parasite/2023067/olm. *Table S1*. List of parasite taxa used in phylogenetic analyses (given in alphabetical order), their hosts, origin, and accession numbers. *Table S2*. Estimates of evolutionary divergence between sequences.
