# Supplementary material for: Hemolivia species infecting Central American wood turtles (Rhinoclemmys pulcherrima manni) and problems with differential diagnosis within the genus Hemolivia
Source: Parasite. 2024 Feb 14;31:1. doi: 10.1051/parasite/2023067 (PMC10865994; doi:10.1051/parasite/2023067)
Supplement: Supplementary file 1 — Table S1. List of parasite taxa used in phylogenetic analyses (given in alphabetical order), their hosts, origin, and accession numbers. [file parasite-31-1-s1.pdf]

Table XX. List of parasite taxa used in phylogenetic analyses (given in alphabetical order), their hosts, origin, and accession numbers.

**Parasite**	**Definitive host, vector**	**Intermediate host**	**Origin**	**GenBank accession number (18S rDNA)**
Goussia ameliae	*Alosa pseudoharengus*	not present	USA (New Jersey)	KP411007
Goussia janae	*Leuciscus leuciscus*	not present	Czech Republic (Soběslav)	AY043206
Haemogregarina balli	*Placobdella* spp.	*Chelydra serpentina serpentina*	Canada (Ontario)	HQ224959
Haemogregarina stepanowi	*Placobdella costata*	*Mauremys caspica*	Turkey (Diyarbakir)	KF992697
Haemogregarina sp. 3133	? (susp. leeches)	*Pelusios williamsi*	Kenya	KF257923
Haemogregarina sp. 3136	? (susp. leeches)	*Pelusios marani*	Gabon	KF257924
Hemolivia cruciata	unknown	*Cuora galbinifrons*	Vietnam (Vính Phúc Province, Melinh Biodiversity Station)	MW514212
Hemolivia mariae 4903	unknown	*Egernia stokesii*	S Australia (Hawker, Flinders Ranges)	KF992711
Hemolivia mariae 4955	unknown	*Egernia stokesii*	S Australia (Hawker, Flinders Ranges)	KF992712
Hemolivia mariae	*Amblyomma albolimbatum*	unknown	W Australia	OL440970
Hemolivia mariae	*Amblyomma albolimbatum*	unknown	W Australia	OL440971
Hemolivia mauritanica GR-9-04	*Hyalomma aegyptium*	*Testudo marginata*	Greece (Volos)	KF992699
Hemolivia mauritanica IQ-4-10	*Hyalomma aegyptium*	*Testudo graeca*	N Iraq (Sulajmáníja region)	KF992700
Hemolivia mauritanica SY-10-10-2	*Hyalomma aegyptium*	*Testudo graeca*	Syria (Qalat Samaan)	KF992701
Hemolivia mauritanica SY-10-10-3	*Hyalomma aegyptium*	*Testudo graeca*	Syria (Qalat Samaan)	KF992702
Hemolivia	*Hyalomma*	*Testudo graeca*	Syria (Qalat Samaan)	KF992703

mauritanica SY-20-10-3	*aegyptium*			
Hemolivia mauritanica SY-28-10-3	*Hyalomma aegyptium*	*Testudo graeca*	Syria (Tele Karamah)	KF992706
Hemolivia mauritanica SY-49-05	*Hyalomma aegyptium*	*Testudo graeca*	Syria (Ayn Dara)	KF992708
Hemolivia mauritanica SY-72-10-2	*Hyalomma aegyptium*	*Testudo graeca*	Syria (Krak des Chevaliers)	KF992709
Hemolivia mauritanica TR-8-08	*Hyalomma aegyptium*	*Testudo graeca*	Turkey (Muş)	KF992698
Hemolivia mauritanica Vendelin	*Hyalomma aegyptium*	*Testudo marginata*	Greece (Platamonas)	KF992710
Hemolivia parvula	tick?	*Kinixys zombensis*	South Africa (KwaZulu-Natal, Ndumo Game Reserve)	KR069082
Hemolivia parvula	tick?	*Kinixys zombensis*	South Africa (KwaZulu-Natal, Ndumo Game Reserve)	KR069083
Hemolivia sp. 1 DJH-2013	*Hyalomma aegyptium*	*Testudo graeca*	Algeria (Ain Chorfa, Guertoufa, Zemmora, Saf Saf, Theniet el Had)	KC512766
Hemolivia sp. voucher MF5504	unknown	*Rhinoclemmys punctularia*	USA (Alabama, Birmingham, Guthrie Turtle Farm)	MN160404
Hemolivia stellata	*Amblyomma rotundatum*	*Rhinella marina*	Brazil (Para, Belem)	KP881349
Hemolivia stellata	*Amblyomma dissimile*	*Rhinella horribilis, Rhinella humboldti*	Colombia (Universidad del Magdalena, Santa Marta, Magdalena)	MH196475
Hemolivia stellata	*Amblyomma dissimile*	*Rhinella horribilis, Rhinella humboldti*	Colombia (Casa Blanca, Santa Marta, Magdalena)	MH196478
Hemolivia stellata	*Amblyomma dissimile*	*Rhinella horribilis, Rhinella humboldti*	Colombia (Universidad del Magdalena, Santa Marta, Magdalena)	MH196480
Hemolivia	*Amblyomma*	*Rhinella*	Colombia	MH196482

stellata	*dissimile*	*horribilis, Rhinella humboldti*	(Universidad del Magdalena, Santa Marta, Magdalena)	
***Hemolivia* sp. NIC-2-13**	unknown	*Rhinoclemmys pulcherrima manni*	southern Nicaragua	
***Hemolivia pulcherrima* sp. n. NIC-6-13**	unknown	*Rhinoclemmys pulcherrima manni*	southern Nicaragua	
***Hemolivia* sp. NIC-9-13**	unknown	*Rhinoclemmys pulcherrima manni*	southern Nicaragua	
***Hemolivia pulcherrima* sp. n. NIC-12-13**	unknown	*Rhinoclemmys pulcherrima manni*	southern Nicaragua	
***Hemolivia pulcherrima* sp. n. NIC-17-13**	unknown	*Rhinoclemmys pulcherrima manni*	southern Nicaragua	
***Hemolivia* sp. NIC-19-13**	unknown	*Rhinoclemmys pulcherrima manni*	southern Nicaragua	
***Hemolivia pulcherrima* sp. n. NIC-27-13**	unknown	*Rhinoclemmys pulcherrima manni*	southern Nicaragua	
***Hemolivia pulcherrima* sp. n. NIC-29-13**	unknown	*Rhinoclemmys pulcherrima manni*	southern Nicaragua	
***Hemolivia* sp. NIC-4-13**	unknown	*Rhinoclemmys pulcherrima manni*	southern Nicaragua	KF992713
***Hemolivia pulcherrima* sp. n. NIC-10-13**	unknown	*Rhinoclemmys pulcherrima manni*	southern Nicaragua	KF992714
Hepatozoon americanum	*Amblyomma maculatum*	*Canis familiaris*	experimental infection at the Oklahoma State University, USA)	AF176836
Hepatozoon ayorgbor	*Culex* spp.	*Python regius*	Ghana	EF157822
Hepatozoon banethi	*Ixodes tasmani*	*Canis familiaris*	Australia (Tasmania, Devonport)	MG758133
Hepatozoon canis	*Rhipicephalus sanguineus*	*Vulpes vulpes*	Spain	AY150067
Hepatozoon canis	*Rhipicephalus sanguineus*	*Canis familiaris*	Sudan	DQ111754
Hepatozoon catesbianae	*Culex* spp.	*Lithobates catesbeianus*	Canada (Ontario)	HQ224963

Hepatozoon felis	*Rhipicephalus* spp.	*Felis catus*	Spain	AY628681
Hepatozoon magna	Culicinae?	*Pelophylax esculentus*	France (Corsica)	HQ224960
Hepatozoon musa	unknown	*Philodryas nattereri*	Brazil (Upanema, Rio Grande do Norte)	KX880079
Hepatozoon sipedon	*Culex* spp.	*Nerodia sipedon sipedon*	Canada (Ontario)	JN181157
Hepatozoon sp.	*Amblyomma aureolatum*?	*Dusicyon thous*	Brazil	AY461377
Hepatozoon sp.	fleas?	*Clethrionomys glareolus*	Spain (Province of Soria)	AY600625
Hepatozoon sp.	*Ixodes*?	*Martes martes*	Spain	EF222257
Hepatozoon sp.	*Ixodes neuquenensis*?	*Dromiciops gliroides*	S Chile (northern Chiloé Island)	FJ719814
Hepatozoon sp.	unknown	*Abrothrix olivaceus*	S Chile (northern Chiloé Island)	FJ719818
Hepatozoon sp.	unknown	*Scelarcis perspicillata perspicillata*	Morocco (Debdou)	HQ734791
Hepatozoon sp.	unknown	*Podarcis vaucheri*	Morocco (Lake Tislit)	HQ734793
Hepatozoon sp.	unknown	*Eumeces algeriensis*	Morocco (Ouazane)	HQ734796
Hepatozoon sp.	unknown	*Atlantolacerta andreanskyi*	Morocco (Oukaimeden)	HQ734798
Hepatozoon sp.	unknown	*Timon tangitanus*	Morocco (Azrou)	HQ734799
Hepatozoon sp.	unknown	*Timon tangitanus*	Morocco (Agoudal)	HQ734801
Hepatozoon sp.	unknown	*Podarcis hispanica*	Spain (Alba de Tormes)	JQ762310
Hepatozoon sp.	unknown	*Podarcis lilfordi*	Spain (Balearic Islands, Cabrera Island))	JQ762311
Hepatozoon ursi	*Haemaphysalis* spp.	*Ursus thibetanaus japonicus*	Japan	EU041718
Karyolysus galloti	unknown	*Gallotia caesaris*	Spain (Canary Islands)	MK396906
Karyolysus cf. *lacazei*	*Ixodes ricinus*	*Lacerta schreiberi*	Spain	MK497254
Karyolysus paradoxa	unknown	*Varanus albigularis*	South Africa (KwaZulu-Natal, Ndumo Game Reserve)	KX011040

Karyolysus sp.	*Ophionyssus* sp.	*Podarcis muralis*	Slovakia (Čabraď)	KJ461939
Karyolysus sp.	*Ophionyssus* sp.	*Lacerta agilis*	Poland (Odolanow)	KJ461940
Karyolysus sp.	*Ophionyssus* sp.	*Lacerta trilineata*	Romania (Sacele)	KJ461942
Karyolysus sp.	*Ophionyssus* sp.	*Lacerta viridis*	Hungary (Godollo)	KJ461943
Karyolysus sp.	*Ophionyssus* sp.	*Zootoca vivipara*	Poland (Odolanow)	KJ461946
Karyolysus sp.	*Ophionyssus* sp.?	*Iberolacerta horvathi*	Slovenia	OK348285
